# Cell cycle dynamics in a response/signalling feedback system with a gap

**DOI:** 10.1080/17513758.2014.904526

**Published:** 2014-04-08

**Authors:** Xue Gong, Richard Buckalew, Todd Young, Erik Boczko

**Affiliations:** ^a^ Department of Mathematics, Ohio University, Athens, OH45701, USA; ^b^ Department of Biomedical Informatics, Vanderbilt University Medical Center, Nashville, TN, USA

**Keywords:** yeast metabolic oscillations, nonlinear feedback

## Abstract

We consider a dynamical model of cell cycles of *n* cells in a culture in which cells in one specific phase (*S* for signalling) of the cell cycle produce chemical agents that influence the growth/cell cycle progression of cells in another phase (*R* for responsive). In the case that the feedback is negative, it is known that subpopulations of cells tend to become clustered in the cell cycle; while for a positive feedback, all the cells tend to become synchronized. In this paper, we suppose that there is a gap between the two phases. The gap can be thought of as modelling the physical reality of a time delay in the production and action of the signalling agents. We completely analyse the dynamics of this system when the cells are arranged into two cell cycle clusters. We also consider the stability of certain important periodic solutions in which clusters of cells have a cyclic arrangement and there are just enough clusters to allow interactions between them. We find that the inclusion of a small gap does not greatly alter the global dynamics of the system; there are still large open sets of parameters for which clustered solutions are stable. Thus, we add to the evidence that clustering can be a robust phenomenon in biological systems. However, the gap does effect the system by enhancing the stability of the stable clustered solutions. We explain this phenomenon in terms of contraction rates (Floquet exponents) in various invariant subspaces of the system. We conclude that in systems for which these models are reasonable, a delay in signalling is advantageous to the emergence of clustering.

## 1. Introduction

### 1.1 Background

We consider a generalization of a dynamical model of the mitotic cell division cycle (CDC) with response–signalling feedback that was introduced by authors in [2,30]. The dynamics of the model are governed by

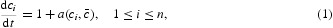

where *c*_*i*_ is the position of the cell within the cycle and 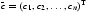 denotes the state of all the cells in the culture. Here 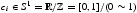, so the phase space we consider is the *n*-torus. The term 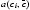 allows for cells in one part of the cell cycle *S* (signalling) to effect the growth/progression of cells in another part *R* (responsive), i.e. feedback. Specifically, we will consider the situations where cells in *S* produce chemical agents that retard the cell cycle progress of cells that are in *R*. Positive feedback from *S* to *R* could also be considered. In previous studies [2,30], we assumed that *R* immediately proceeds *S*, that is, they intersect at a boundary, such as the boundary between the G1 and S phases in yeast. In this manuscript, we explore the modelling assumption that there is a gap between the two regions. A gap is biologically justified by the inevitable delay between the onset of production of a potential signalling agent and its effectiveness.

We are motivated by yeast metabolic oscillations (YMOs), a phenomenon that has been observed in experiments for at least 60 years [10,16,18] and remains a topic of intense interest [5–8,12,19–24,27–29,31]. A link between YMO and the cell division cycle (CDC) was noticed as early as in [16,18], but this connection seems to have been largely ignored. However, in [15,27] a connection between YMO and CDC was again noted in genetic expression time series.

Bockzo *et al.* [2], based on data in [23], proposed *cell cycle clustering* as a possible explanation of the interaction between YMO and the CDC. By clustering we mean groups of cells traversing the CDC in near temporal synchrony, not spatial clustering (cultures that exhibit YMO occur in well-mixed bioreactors). In [2,30], we studied both simple and general forms of (1) with the hypothesis that cells in one part of the CDC may influence the growth or CDC progression of cells in other parts of the CDC through diffusible metabolites. We showed analytically and numerically that CDC feedback such as this can robustly cause CDC clustering in the models. For example, a significant subpopulation of cells in the critical S-phase might affect metabolism production and the metabolites may in turn inhibit or promote cell growth in the later part of the G1 phase, thus setting up a feedback mechanism in which YMO and CDC clustering are inextricably intertwined. In [4], a model with an explicit term to represent the signaling agent is studied.

Guided by these mathematical results, we verified the existence of clusters in two types of oscillating yeast cultures using both bud index and cell density data [2,26]. Experimental evidence in [24] also supports the existence of clusters in some YMO experiments. Since *Saccharomyces cerevisiae* is a model organism, it is important to understand the interconnectedness of the CDC and metabolism [1,6,15,24,27,29]. Also, yeast are used in many bio-engineering processes and understanding their metabolism and cell cycle is of interest in some applications [14,25,31].

In the rest of the paper, we will use 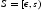 to denote the signalling region, *R*=[*r*, 1) to denote the response region, and ε to denote the width of the gap, [0, ε], between *S* and *R*. The gap is intended to mimic a delay of the chemical signalling agents responsible for the feedback.

For these modelling assumptions to be quantitatively realistic, there need to be some estimate of the size of the gap. One of the metabolites that is known to have effects on cell cycle progression and is thought to be the primary signalling agent in glycolytic oscillations is acetaldehyde [8]. The diffusion rate of acetaldehyde across a yeast cell membrane is estimated to be *d*=300 s^−1^ [8], so this rate is very fast. The diffusion/convection in the well-mixed bioreactor is even faster. The decay rate for acetaldehyde in the same yeast bioreactor study was estimated to be 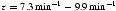 [8]. In this experiment, the yeast were performing fast glycolytic oscillations so the production rate of acetaldehyde would need to be on the same order of magnitude. This implies that time scale of a delay is on the order of 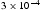 in the normalized coordinates. The glycolytic oscillation in that experiment had a period of 37 s. Using this as an upper bound of the time scale of acetaldehyde production and diffusion would give us an upper bound on the gap of 0.0025. It is not known with certainty that acetaldehyde is the only or even the dominant signalling agent in the oscillations where clustering has been observed. Thus at this point these estimates are only speculative. Fortunately, as we shall see, we are able to analytically treat a broader range of gaps.

### 1.2 The feedback model

The following is an RS-feedback with a gap system (compare [30]):

Definition 1.1 Consider *n* cells whose coordinates are given by *c*_*i*_∈[0, 1], 1 identified with 0. When a cell reaches 1 it continues at 0. We call such a system an RS-feedback system with ε gap if:

(H1) *R* is an interval that precedes another interval *S* with a small ε length gap, i.e. the distance between the last endpoint of *R* and the first endpoint of *S* is ε;
(H2) 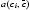 vanishes except when *c*_*i*_∈*R* and there are some *c*_*j*_ in *S*;
(H3) 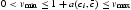 for all *c*_*i*_ and 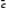;
(H4) Feedback is monotone – adding a cell to *S* will increase the value 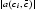 for all *c*_*i*_∈*R*; and
(H5) 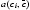 is a smooth function for *c*_*i*_ in the interior or *R* and each *c*_*j*_ in the interior of *S, j*≠*i* and the one-sided derivatives exist at the boundaries of *R* and *S*.

A positive feedback will mean that *a* is positive for *c*_*i*_∈*R* when at least one *c*_*j*_ is in *S*. We define a negative feedback analogously.

Hypothesis (H1) specifies the signalling, the response, and the delay in signalling. The meaning of Hypothesis (H2) is that cells will not experience any feedback, unless it is caused by cells in the signalling region. Hypothesis (H3) assumes that feedback is bounded, i.e. there are upper and lower limits on the rate of progression of a cell in the cycle. Note that we are not allowing cell cycle arrest, a common biological state. Hypothesis (H4) supposes that feedback must increase as the number of signalling cells increases. We note that hypotheses (H3) and (H5) (a technical assumption) are sufficient to imply existence and uniqueness of solutions of (1) [30].

Recall that we specify the coordinates throughout the paper as follows:

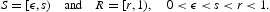

The final endpoint of *R* is 1, which corresponds to 0, the initial endpoint of the ε gap [0, ε).

In this paper, we will mainly study the following RS system with a gap:

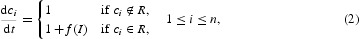

where

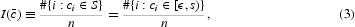

i.e. *I* is the fraction of cells in the signalling region. The ‘response function’ *f*(*I*) in Equation (2) must satisfy *f*(0)=0 and be monotone (H4), but can be non-linear. These assumptions correspond to the suppositions that no cell in *S* implies no feedback is exerted and that more cells in *S* must result in more feedback on cells in *R*.

It is known in cell cycle models *without feedback* that the inclusion of small dispersive or diffusive forces leads to asynchronous population profiles in which a ‘steady-state’ profile is asymptotically stable [9,11]. Our previous work shows that this is not the case when feedback is included, but rather populations tend to form temporal clusters. There are other recent discoveries of clustering behaviour due to a negative feedback, especially in phase oscillator models of networks of neurons [13,17].

## 2. Dynamics of clusters via return maps

In the remainder of this manuscript, we consider the existence and stability of periodic ‘clustered’ solutions. Because the cells are assumed to be identical and obey (1), cells that initially share the same coordinate will remain synchronized as time evolves. By a *cluster*, we formally mean a group of cells that have identical coordinates in the CDC.

Because clustered solutions will remain clustered, one may consider such solutions using the coordinates of the clusters rather than those of individual cells. In doing so, one may greatly decrease the dimensions of the system. In a yeast bioreactor culture, *n* can be in the order of 10^10^, while we may study clustered solutions with a small number of clusters.

For example, if the full system satisfies Equation (2), then the equation of progression for *k* clusters consisting of an equal number of cells is

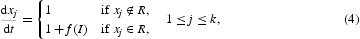

where *I* is the fraction of clusters in the signalling region: 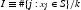.

The extreme case of clustering is if all cells have identical coordinates; then we have a single cluster and Equations (4) will become a single equation. It will be trivial since there will never be a feedback acting on the clusters. The only solution is a periodic solution that runs at velocity 1 around the circle (i.e. 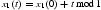 for all *t*>0). We refer to this special clustered solution as *synchronized*.

We note in [2] that a synchronized solution is locally asymptotically stable for a positive feedback and unstable for a negative feedback in the general model (1). This is because when a group of cells that are nearly synchronized leaves *R* and enters *S* the first cells in the group exert feedback on the trailing cells. For a positive feedback, the trailing cells in *R* will speed up. The cells will eventually approach synchronization. For a negative feedback, cells in *R* will slow down instead, thus moving further from the synchronized state.

In contrast, with a gap between the responsive region *R* and the signalling region *S*, the above situation will not happen. If a solution consists of all cells within ε of each other, then when this group of cells crosses from *R* to *S*, all of the cells will be out of *R* before the first one enters *S*. Hence, no cells in the solution experience feedback and the following proposition is obtained.

Proposition 2.1 For the most general RS feedback with a gap (1) and Definition 1.1 with either a positive or a negative feedback, the synchronized periodic solution is (locally) neutrally stable.

It is natural to ask whether or not the inclusion of a gap also changes the global dynamics of the system. As discussed in [30], we can assume that all coordinates *x*_*j*_(0) of the *k* clusters are initially well-ordered as

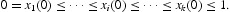

This ordering is preserved under the dynamics (in a sense that can be made precise) and the first coordinate *x*_1_ must eventually reach 1, i.e. there exists *t*_R_ such that 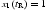. Thus, the set *x*_1_=0 defines a Poincaré section for the dynamics and the mapping

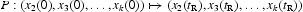

defines the corresponding return map.

Starting from *t*=0, we compute the time *t*_1_ that *x*_*k*_ needs to reach 1 and the location of the remaining clusters at this time. As in [30] we define a map *F* by

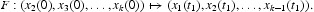

**Fig. 1.**
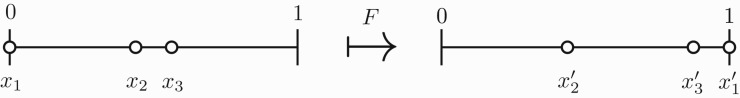
An illustration of the map *F* with *k*=3; *F*(*x*_2_, *x*_3_)=(*x*′_2_, *x*′_3_).

Note that 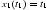 by the assumptions. If the clusters are identical to each other, then the map *F* is a root of the Poincaré map, i.e. *F* ^*k*^=*P* and it can be regarded as a continuous, piecewise smooth map of the (*k*−1)-dimensional simplex

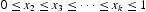

into itself. See Figure 1.

We concentrate on studying periodic clustered solutions that satisfy:

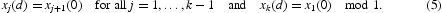

We will refer to such solutions as *k*-*cyclic* clustered solutions. If *k* is a divisor of *n*, then a *k*-cyclic solution exists consisting of *n*/*k* cells in each cluster. This fact can be proved using the Brouwer fixed point theorem applied to *F* [30]. As pointed out in [3], clustered solutions become less and less important as *k* grows larger.

In the remainder of this manuscript, we will use *k* to denote the number of clusters and will suppose that each cluster consists of *n*/*k* cells.

## 3. Analysis of *k*=2 cluster systems

In this section, we will compute and analyse the map *F* for two equal clusters in the model (4). If we start *x*_1_ at 0 and integrate until *x*_2_ reaches 1, then calculating *F* reduces to solving:

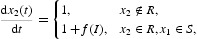

where 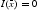 or ½, because there can be only one cluster in the signalling region *S* when another cluster is in the response region *R*. Let 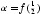.

We will see that the problem can be analysed by dividing it into the following two cases:

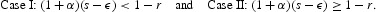



### 3.1 Case 1: (1+α)(*s*−ε)<1−*r*

There are five situations depending on the location of *x*_2_:

(a) 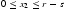. In this case, *x*_1_ leaves *S* before *x*_2_ enters *R*, thus *x*_2_ is not submitted to any feedback and 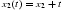 for all *t* in [0, 1−*x*_2_]. Hence, 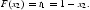

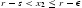
. Because *x*_2_ enters the responsive region [*r*, 1) after *x*_1_ gets into [0, ε) and before *x*_1_ leaves 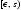, *x*_2_ is influenced by *x*_1_ for some part of the time when it is in the responsive region. We can obtain the following piecewise-defined function for the position of *x*_2_ depending on time:

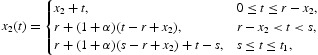

where *x*_2_(*t*_1_)=1, so we find 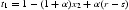. Hence, 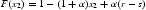.

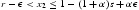
. In this case, *x*_2_ is influenced by *x*_1_ for the entire time when *x*_1_ is in the signalling region. This is because *x*_1_ arrives at *S* after *x*_2_ reaches *r* and *x*_1_ gets out of *S* before *x*_2_ reaches 1. We may obtain:

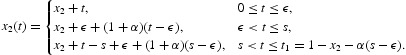

Then 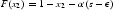.

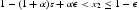
. In this case, *x*_2_ reaches *r* before *x*_1_ gets into *S*, and when *x*_2_ reaches 1, *x*_1_ is still in *S*. We can find the position of *x*_2_:

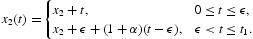

We can find that 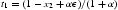 by *x*_2_(*t*_1_)=1. Thus, 
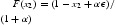
.

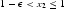
. In this case, *x*_1_ does not affect *x*_2_ because *x*_2_ reaches 1 before *x*_1_ enters *S*. So, 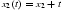 for all 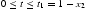, then 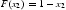.

Combining these five sub-domains, we can obtain the map *F* for Case I:

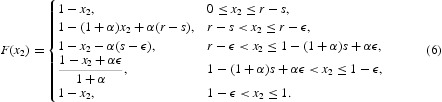

### 3.2 Case II: (1+α)(*s*−ε)≥1−*r*

Similar to the computation above, we consider five subcases:

(a) 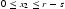. Same as Case 1(a), we have 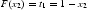.

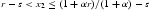
. In this case, *x*_1_ reaches ε first, then *x*_2_ enters *R*, and *x*_1_ reaches *s* before *x*_2_ reaches 1. So *x*_2_ is partially influenced by *x*_1_. This yields the position of *x*_2_ expressed by the function:

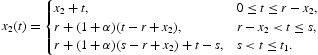

Hence, we can derive 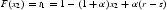 by solving *x*_2_(*t*_1_)=1.

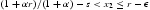
. Note that 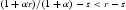 is implied by this case. Here *x*_2_ is influenced by *x*_1_ for the entire time when it is in the responsive region because *x*_2_ enters *R* after *x*_1_ gets into *S* and when *x*_2_ reaches 1, *x*_1_ is still in *S*. We obtain the following piecewise-defined function for the position of *x*_2_:

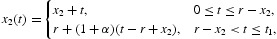

where 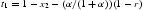. Hence, 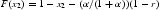.

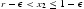
. For this case *x*_2_ enters *R* before *x*_1_ reaches ε, and so it is influenced by *x*_1_ for some part of the time when it is in the responsive region until it reaches 1.

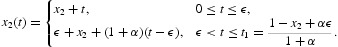

Thus, 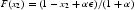.

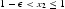
. This is the same as Case 1(e) that *x*_1_ does not affect *x*_2_. We obtain: 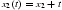 for all *t*∈[0, 1−*x*_2_]. Thus, 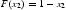.

We combine the five subcases and arrive at the map *F* for Case II:

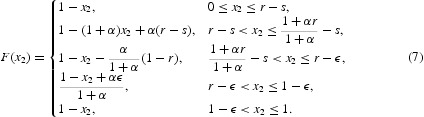

### Analysis of the dynamics

**Fig. 2.**
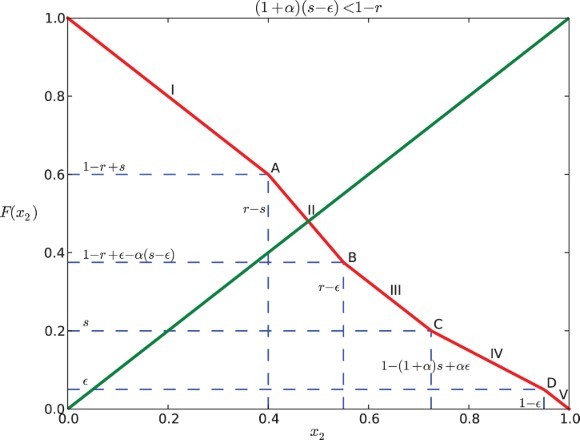
Return map *F* for Case I when α=0.5, *s*=0.2, *r*=0.6, and ε=0.05.

**Fig. 3.**
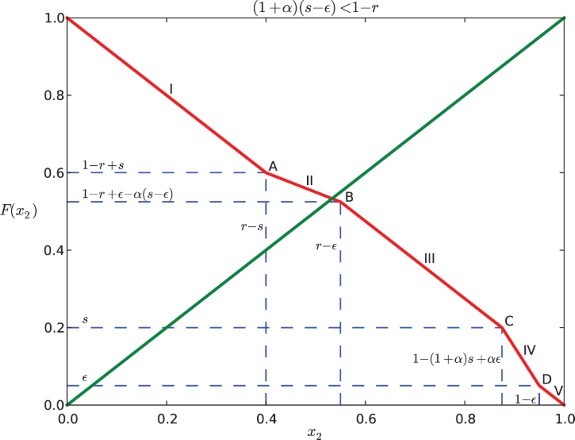
Return map *F* for Case I when α=−0.5, *s*=0.2, *r*=0.6, and ε=0.05.

**Fig. 4.**
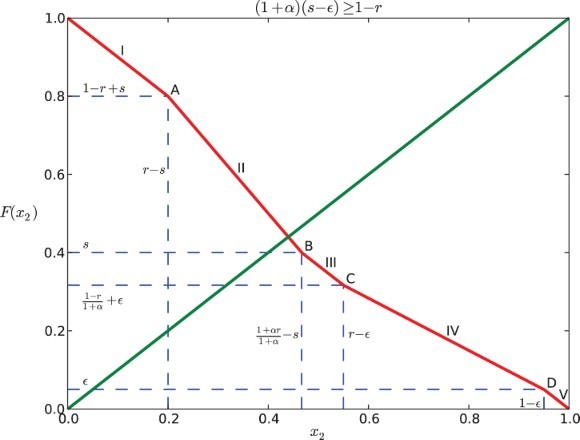
Return map *F* for Case II when α=0.5, *s*=0.4, *r*=0.6, and ε=0.05.

**Fig. 5.**
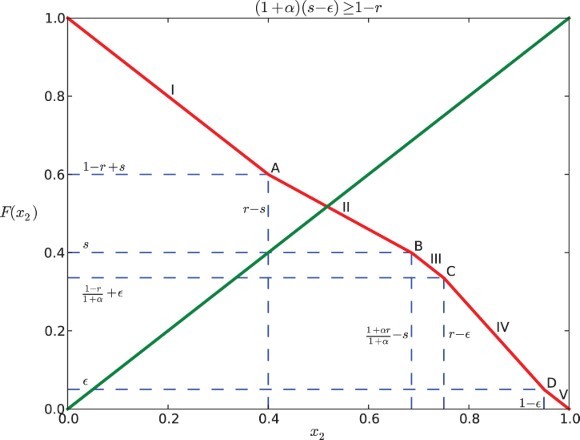
Return map *F* for Case II when α=−0.3, *s*=0.4, *r*=0.8, and ε=0.05.

The graph of *F* coincides with the anti-diagonal *y*=1−*x*_2_ for 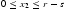 and 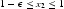 (Figures 2–5). For a positive feedback 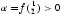, it is strictly less than 1−*x*_2_ when 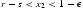 (Figures 2 and 4); while for a negative feedback α<0, it is strictly greater than 1−*x*_2_ if 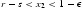 (Figures 3 and 5). In the following discussion, we consider the restriction that 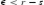 is relatively small.

For Case I (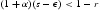), it is seen that

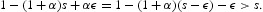

Therefore, the graph of *F* cannot intersect 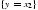 at the line segments IV and V or the points C and D. This is illustrated in Figures 2 and 3.

Similarly, for Case II (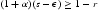), we can find that the graph of *F* and the diagonal cannot intersect at C, IV, D or V (see Figures 4 and 5), because

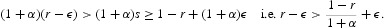



Considering the above situations, we conclude the following:

(1) If 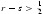, the intersection of the graph of *F* and the diagonal *y*=*x*_2_ lies in the first line segment (0<*x*_2_<*r*−*s*), denoted as I. Each point of the interval [1−*r*+*s, r*−*s*] is part of a 2-periodic orbit 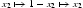, except for the point 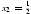 which is fixed. Hence, the return map *F* ^2^ has an interval of neutrally stable fixed points centered around 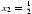.
If 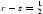, the intersection, denoted as A is at 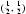. It is a unique fixed point that is stable for negative α and unstable for positive α.
If 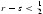, then 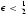, thus 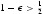. There are three possibilities depending on where the diagonal line *y*=*x*_2_ intersects the graph of *F*. Also here, we need to consider Case I and Case II separately.
  (a) If the diagonal intersects the second line segment (II), the intersection lies in the interval 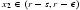 for Case I or 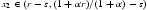 for Case II. Hence, there is a unique fixed point. It is stable if α<0 and unstable if α>0.
  If the diagonal hits the boundary between segments II and III of *F* (point B as in the figures), there is a unique fixed point at 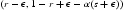 for Case I or at 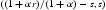 for Case II, which is stable for negative α and unstable for positive α.
  If the diagonal intersects the third segment (III) of the graph of *F*, since the slope is −1, there is an interval of neutral period 2 points containing in 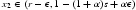 for Case I or 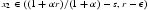 for Case II. The edge of the interval is stable for negative α and unstable for positive α.

Note that in all cases, the 2-cyclic solution corresponds to the unique fixed point of *F*. According to the above results, we find four distinct types of dynamics; two for positive feedback and two for negative feedback.

- Positive feedback:
  (a) The 2-cyclic solution is unstable when the intersection is at II, A or B.
  The 2-cyclic solution belongs to an interval of fixed points of period-2 solutions when the intersection is at I or III. This interval is repelling.
- Negative Feedback:
  (a) The 2-cyclic solution is stable when the intersection is at II, A or B.
  The 2-cyclic solution belongs to an interval of fixed points of period-2 solutions when the intersection is at I or III. This interval is attracting.

These behaviours are exactly the same as for the model with no gaps [30].

Furthermore, we note that in the parameter regions where the fixed point of the map is either stable or unstable, the slopes of *F* are *exactly the same as for the model without gaps* (see [30]).

## 4. Cyclic *k*=*M*+1 cluster solutions

### 4.1 Isolated solutions

Denote |*S*| as the length of the interval 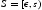, which is *s*−ε and |*R*|=1−*r* denotes the length of the interval *R*=[*r*, 1). We say that a cluster of cells is *isolated* if there are gaps between the cluster and any other cells on either side of length at least 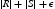 and *strictly isolated* if the widths of gaps are more than 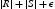. Strictly isolated clusters cannot exert feedback on cells outside the cluster, or have feedback exerted upon them from outside.

We consider the case only when ε is small, i.e. 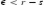, as in the former section. The following definition will play a large role in the analysis of the model.

Definition 4.1 Define

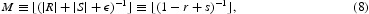

i.e. *M* is the maximum number of isolated clusters that can simultaneously exist.

We will consider the cyclic solutions consisting of *k*=*M*+1 clusters. For *k*=*M*+1, it can be shown that the cyclic *k*-cluster solution has initial conditions: 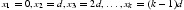. During each time interval *d*, each cluster will move to the position of the cluster ahead of it.

For *k*=*M*+1 and for all solutions in a neighbourhood of the cyclic solution above, at most one cluster can be in the signalling region *S* at a time. Thus, the feedback experienced by any cluster in *R* is either 0 or *f*(1/*k*). Hereafter, when studying systems with *k*=*M*+1 clusters, we will use the abbreviation:

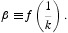



### 4.2 Order of events for the cyclic solution

The evolution of a solution can be described in terms of the *order of events* of the solution. Clusters of cells progress through the cell cycle at rates specified by Equation (2). These rates remain constant until a cluster reaches ε, *s, r*, or 1. Given the next such event and the index *j* of the cluster for which it occurs, we can calculate the time elapsed until the event occurs by finding out the initial position of cluster *j* and its corresponding rate during that time. We will use 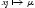 to denote the event that cluster *j* reaches the milestone μ. Then for the order of events, for example, as in Case I below the notation 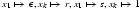 means that first *x*_1_ reaches point ε, then *x*_*k*_ reaches *r*, etc.

We discuss in the following cases the possibilities of the orders of events for the cyclic solution.

*Case* I: *x*_2_=*d*>*s* and 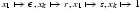.

By calculating the position of *x*_*k*_ in each step, we have

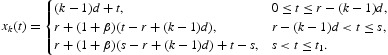

Here, *x*_*k*_(*t*_1_)=1. So, 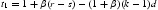 (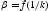). At time *t*_1_, *x*_1_ reaches *x*_2_=*d* (since it is moving with speed 1). Thus,

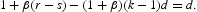

One can solve for *d*:

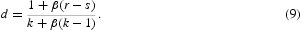

This case can only happen when *d*>*s* and 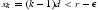. Using Equation (9) in these two inequalities gives conditions on *r* and *s* required by this order of events:

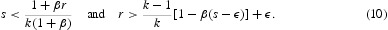

*Case* II: *x*_2_=*d*>*s* and 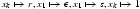.

Similar to Case I, we may calculate:

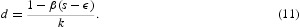

The above *d* also needs to satisfy *d*>*s* and 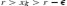, so we obtain:

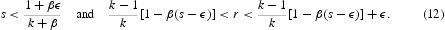

*Case* III: *x*_2_=*d*>*s* and 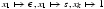.

This order of events can happen only when *x*_*k*_>*r*. We can find that *d* is the same as in Equation (11), 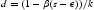, and the order of events in this case implies the inequalities:

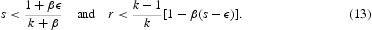

*Case* IV: *x*_2_=*d*<*s* and 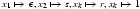.

After some calculation of the final position of *x*_1_, we find that

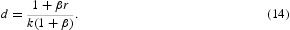

This case occurs under the conditions that 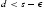 and *x*_*k*_<*r*. Using Equation (14) with the order of events gives that

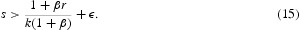

*Case* V: 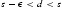 and 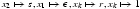.

After a similar calculation, we find the same *d* as in Equation (14) that is 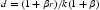. Applying this result to 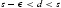 and 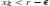, we obtain the following conditions for Case V to occur:

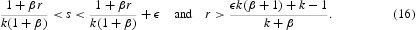

*Case* VI: 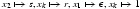.

This case occurs if and only if *d*<*s* and 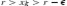. Solving for *d*, we find that

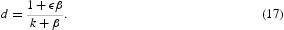

We substitute Equation (17) into the above inequalities to obtain:

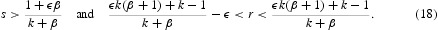

**Fig. 6.**
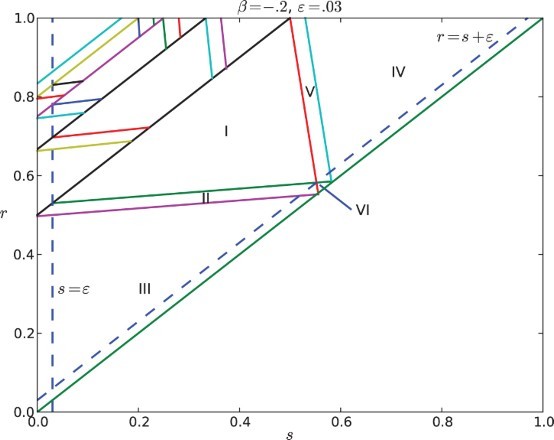
Regions of parameter space for the *k* cyclic solutions with *k*=*M*+1. Here the feedback is negative with β=−.2 and the gap is ε=0.03. The widest diagonal band contains the parameters for *k*=2 and is partitioned into six cases. Note that case VI disobeys the constraint ε<*r*−*s*. The rest of the diagonal bands contain parameters for *k*=3, 4, … and each of them is partitioned into five cases.

The regions described by the inequalities in Equations (10)–(18) are illustrated in Figure 6. We can observe that cases I–VI exhaust the parameter set in (*r, s*) and there is never more than one cluster in the signalling region when *R* is non-empty. Thus the dynamics of the system are determined by the feedback 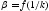.

### 4.3 Stability of the cyclic solution

Now we will calculate the return map *F*. We note that *F* is affine in a neighbourhood of the cyclic solution provided that the parameters are in the interior of one of the cases. Thus, 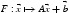 where 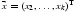 where *A* is a matrix. To find the stability of the fixed point, the linear part of the system, i.e. *A* needs to be analysed.

In Case I, we can calculate the time for *x*_*k*_ reaching at 1, that is 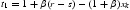. Since each of the clusters moves for time *t*_1_, we can find 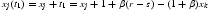 for 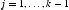. Given that *x*_1_=0 and *x*_*k*_(*t*_1_)=1, we have 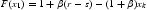 and *F*(*x*_*k*_)=1. Thus we can obtain the matrix *A* by taking the partial derivatives of the return map 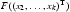:

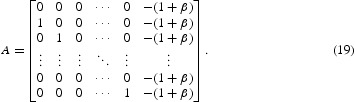

In [30], it was proved that all the eigenvalues of *A* defined by Equation (19) lie outside of the unit disc for β>0 and lie on the interior of the unit disc for β<0. Thus, the fixed point of the return map *F* in Case I is stable for a negative feedback and unstable for a positive feedback, so is the *k*-cyclic solution.

In Cases II and III, following the same procedures as above, we can calculate that 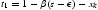. Thus, 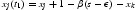 for 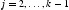, 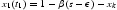, and *x*_*k*_(*t*_1_)=1. Therefore, the linear part of the map at the fixed point is represented by the matrix:

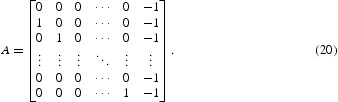

In Cases IV and V, we can find that 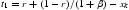. Here the coefficient for the *x*_*k*_ term is still −1, so the linear part of the map is the same as the matrix *A* given by Equation (20) in cases II and III. It is well known that the eigenvalues of this matrix *A* all have (complex) modulus 1. Thus, the *k*-cyclic solution is neutrally stable in all the cases from II to V.

For Case VI, the time for *x*_*k*_ to arrive at 1 is 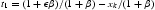. Note that this case only occurs for *M*=1 or *k*=2 and 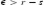. For *M*>1 and 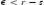, this region is outside the band *k*=*M*+1. The matrix *A* can be derived as follows:

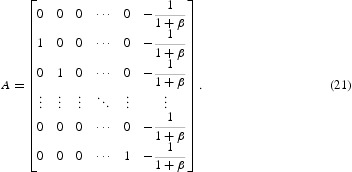

We can use a change of variable to study the stability of this return map compared in the result for Equation (19). We conclude that the fixed point is stable for a positive feedback and unstable for a negative feedback in this case.

The dynamics for the *k*=*M*+1 cyclic solutions can be summarized as follows:

(1) When 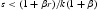 and 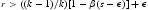, the *k*-cyclic solution is stable for a negative feedback and unstable for a positive feedback.
For *k*=2 and 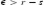, when 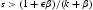 and 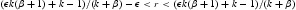, the *k*-cyclic solution is stable for a positive feedback and unstable for negative feedback.
Otherwise, the *k*=*M*+1-cyclic solution is neutrally stable.

The behaviour in Equation (2) occurs only on a small part of parameter space with 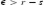, i.e. Case VI in Figure 6.

The behaviour in Equations (1) and (3) is entirely consistent with those for the model without a gap [30]. Further, as noted above, the matrix *DF* in the stable and unstable cases are *exactly the same* as for the model without a gap. Thus, the gap does not even alter the strength of the stability or instability; the linearization of the return maps is exactly the same.

We note that the region in parameter space for which the *k*=*M*+1 cyclic solution is stable is exactly the same as for the model without a gap, except the strip of width ε representing Case II is removed.

## 5. Numerical simulations

### 5.1 Observed clusters

We simulated *n*=420 cells for the model with and without a gap of 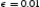. It was studied that clusters form under negative feedback and the exact form of the feedback function does not matter for the general RS feedback system in [30]; thus, in all the simulations we set the feedback function to be *f*(*I*)=−*I* for simplicity. We used a first-order Euler integration with an adaptive step size so that the step matched intervals on which the vector field is constant. By doing this there is no truncation error and all calculations are locally accurate to machine precision.

**Fig. 7.**
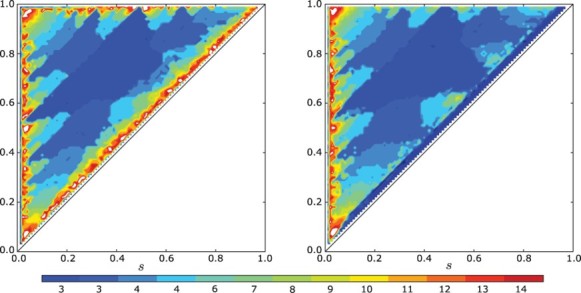
The number of clusters realized in a cell cycle simulation (a) with no gap and (b) with a gap of ε=0.05 when *s*≥0.01 and ε=0.5 *s* when *s*<0.1. In both plots, the feedback is linear and negative with *f*(*I*)=−*I*. Note the strong agreement between the two plots.

**Fig. 8.**
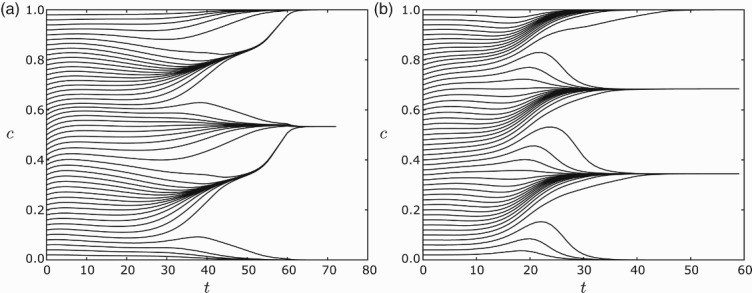
Clustering of *n*=120 equi-distributed cells.(a) For *s*=0.2, *r*=0.6 and ε=0.03, the cells converge to two clusters. (b) For *s*=0.2, *r*=0.8 and ε=0.01, the cells converge to three clusters. For all simulations the feedback was taken to be *f*(*I*)=−*I*. Note in (a) that they first appear to form four clusters, but merge into two clusters.

In Figure 7, we show the number of clusters that appear in simulations of the model with and without a gap. For each pair of parameter values (*s, r*), we started with a equi-distributed initial condition and integrated for 100 cell cycles. The number of clusters were counted using a variation of a standard clustering algorithm DBSCAN. One can see that the main features of the plot are unchanged by the addition of a small gap. Note that in both plots, for all but a narrow set of (*s, r*) values around the perimeter, clusters always emerge. We visually inspected a large selection of simulations and observed that in all instances where the algorithm indicated clustering, the solutions were in fact unambiguously clustered. Figure 8 gives some simulation results showing how clusters emerge.

As predicted in Sections 3 and 4, the global dynamics with and without a gap are qualitatively the same.

### 5.2 Speed of convergence

**Fig. 9.**
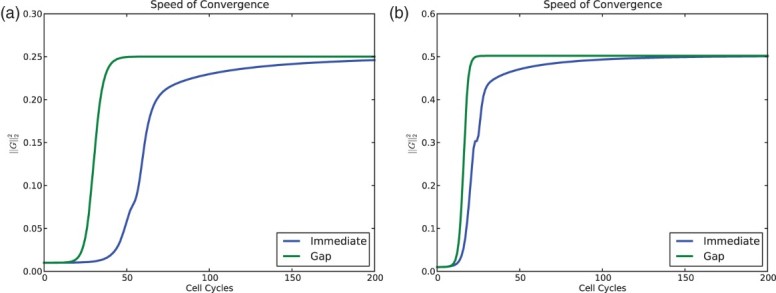
Comparison of convergence speed for the model with and without a gap of ε=0.01 and *n*=60 cells. Norm squared of the gap vector *G* vs. time. (a) For *s*=0.2 and *r*=0.9 both models converge to four clusters. (b) For *s*=0.3 and *r*=0.7 both models converge to two clusters. For all simulations the feedback was taken to be *f*(*I*)=−*I*, and the initial condition was evenly distributed – the global minimum for 0. The model with gaps converges to the clustered solutions much more quickly.


In completing the experiments for Figure 7, we noticed that despite the prediction that the global dynamics be the same, the simulations with a gap tended to converge to the periodic orbits more quickly than without a gap. In Figure 9, we study the speed of convergence to clustered solutions in simulations with a negative feedback. Again we started with evenly distributed initial condition. In order to study the convergence, we used the *vector of gaps*:

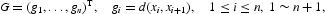

where *d*(*x, y*) is the distance from *x* to *y* on the circle in the direction of the orientation. The Euclidean norm squared, 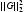 is a measure of how clustered a solution is. It has a global minimum when the cells are equi-distributed around the circle and a maximum (of 1) when all the cells have the same location (synchronized). In the simulation, *G* was measured at the end of each cycle on the Poincaré section 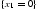.

For the models with and without a gap, we see that solutions converge to a stable clustered configuration. For the model with even a modest gap of 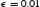 this convergence is much more rapid. This seems to contradict the conclusions of Sections 3 and 4, where we observed that stable cyclic solutions have exactly the same Floquet exponents for the gap and non-gap models. However, the calculation in Sections 3 and 4 were completed in the *clustered subspace*, not in the full phase space of *n* cells. In the case of a negative feedback, where clustered solutions tend to be stable, the model without a gap has a local linear instability effect on individual clusters as they cross the *R*–*S* boundary. In the gap system, this effect does not occur as reflected in Proposition 2.1. The Floquet exponents in the full phase space are more negative with a gap since individual clusters are not locally unstable.

To take a close look at this, we consider a small perturbation of the cyclic solution, close enough to the cyclic solution so that the order of events for those new groups of points does not change (i.e. if one cluster leaves *S* before another enters *R*, after dispersion the two clusters and viewing them as two groups of cells, we now have all cells in one group leave *S* before any cells in the second group enter *R*). For the *k*-cyclic solution, we denote each group by 1, 2, 3, … , *k*. Let time run until the first cell in group *k* reaches at *r* and denote the diameter of the *k*th group at this time to be δ. Then when this group has entirely entered *R*, its diameter has reduced to 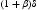 because each cell in the group is subjected to negative feedback β since another group of cells has been in *S* during this time.

For the model without a gap, let time run until the first cell of the *k*th group lies on 1. Because cells which have reached 1 will travel with velocity 1, the diameter of this group after it passes 1 entirely will be the amount of time it takes for the cell lying at 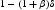 (i.e. the last cell in this group) to reach at 1. To find the maximum of this diameter, we consider the worst case that one cell lies at 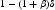 and all other cells of this group are at 1. We can easily calculate that the time it takes for the one cell at 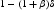 to arrive at 1:

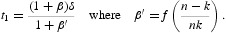

Thus, the diameter of the group is 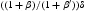. *f* above is the feedback function and the fraction inside is obtained by calculating the proportion of cells inside *S* during this time. By monotonicity of *f*, for negative feedback, 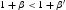. Therefore, the diameter for the *k*th group is strictly less than δ. We can conclude that after each cycle, the diameter δ of a cluster may shrink to 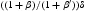.

For the model with ε-gap, we may assume that 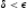. When the last cell in the *k*th group reaches at 1, the rest of the cells in the group still lie inside [0, ε], thus the feedback remains the same. Therefore, the diameter of the group shrinks from δ to 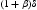 for a negative feedback. Obviously, 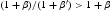 for −1<β<0, and in fact the contraction factor in the worst case for the system without a gap will be very close to 1. This shows that the spectral radius of the linearization for the no-gap system is much closer to 1 than the system with a gap. Thus the maximum eigenvalue of the linearization is smaller for the system with a gap.

The differences in the (local) Floquet exponents account for the marked differences in the convergence once the solution is in a neighbourhood of the clustered solution. These differences are apparent in Figure 9.

It can also be seen in Figure 9 that with a gap, solutions also reach a neighbourhood of the attracting periodic orbit much more rapidly. Generally speaking this can be due to either (1) increased rates of repulsion at repellers (probably unstable periodic orbits) or (2) global factors. The first of these might be amendable to analysis while the second is very difficult to assess.

### 5.3 Simulations with (relatively) large gaps

Note that the condition 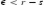 is assumed in Sections 3 and 4. This condition states that:

The gap going from *R* to *S* is smaller than the gap going from *S* to *R*.

Thus if the condition fails, then we are dealing with a fundamentally different kind of system; a system in which *S* could be viewed as located ahead of *R* rather than vice versa.

One situation where the condition is violated is when ε is small, but *r*−*s* is even smaller. We encountered this in Case IV in Section 4, where the dynamics were essentially reversed; the clustered solutions become unstable. In Figure 7, we also observed the violation of the condition for parameter values near the diagonal line *r*=*s*. There we observe synchronous solutions.

**Fig. 10.**
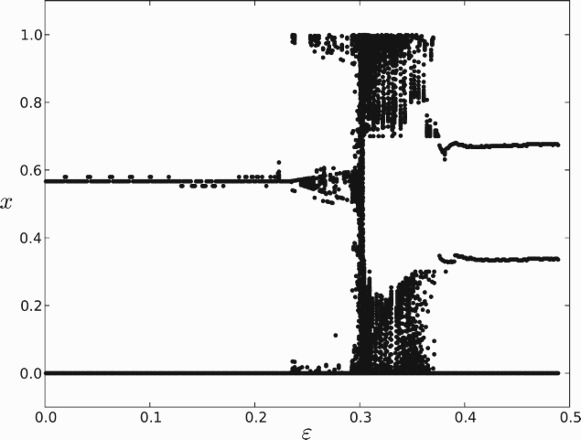
The final configuration of cells in systems with gap sizes varying in [0.1, 4.9].

In order to shed more light on the condition 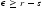, we performed several numerical experiments. In Figure 10, we show the final position of 40 cells after integrating until the system stabilizes to a periodic solution or until a maximum number of steps is reached. Here we set *s*=0.5 and *r*=0.8 and let ε vary on the interval [0.01, 0.49]. When 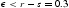, two clusters emerge as we expect from earlier analysis. As ε approaches *r*−*s*=0.3 the stability of the 2-cluster solution weakens and at 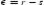 there appears to be a bifurcation. At the critical value 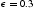, where *R* and *S* are equal in size and exactly opposite one another, no clustering occurs at all, and near that value there appears to be complex and unpredictable behaviour. When ε is approximately 0.37, three clusters emerge. We note that this value corresponds to 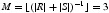, so that it might be possible for three clusters to circulate without interacting. However, a closer look at the numerics reveals that the clusters are in fact interacting and are asymptotically stable with respect to small perturbations. Thus, we conclude that beyond the parameter domain of 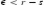 the dynamics exhibit completely different characteristics that would require further analysis to understand.

## 6. Discussion

Inclusion of a gap in the cell cycle system is a reasonable biological modelling assumption. We have found that inclusion of the gap does not complicate the model beyond amenability to do rigorous analysis.

We have fully studied the dynamics of this system when the cells are arranged into two cell cycle clusters. We have shown that the dynamics in this subspace are almost the same as for the model without a gap. The only difference is that the ε-gap introduces a corresponding ε interval of neutral solutions near the synchronous solution. Because this solution has no basin of attraction beyond itself, it is not likely to be realized in real systems.

We also considered the stability of cyclic periodic solutions with *k*=*M*+1 clusters; just enough clusters to guarantee that interactions occur between consecutive clusters. For these cases, we again find that the dynamics mirror those of the system with no gap.

Simulations support these analytical conclusions. We find that the inclusion of a small gap does not greatly alter the global dynamics of the system. Across a wide set of possible parameter values, solutions tend exactly to the same clustering configurations for the gap and no-gap systems.

Thus in the gap model, like the less realistic model with no gap, we find that there are large open sets of parameters for which clustering is a stable phenomenon. We conclude that one should expect to see clustering in some biological systems.

On the other hand, simulations reveal that inclusion of the gap significantly enhances the stability of the stable cyclic solutions and significantly increases the rates at which solutions converge to a stable clustered solution. In simulations the rate of convergence was around twice as fast. Analysis reveals that the gap increases the local stability of a stable cyclic solution.

For a phenomenon to be observable in biological systems, it need not only be stable, but it must be realizable on a time scale that is biologically realistic. These results show that a delay in signalling actually favours clustering.
